# (*E*)-2-Meth­oxy-9-(2-meth­oxy-9*H*-xanthen-9-yl­idene)-9*H*-xanthene

**DOI:** 10.1107/S1600536813017297

**Published:** 2013-06-29

**Authors:** Xiang-Yu Tian, Qin-Hua Song

**Affiliations:** a Department of Chemistry, University of Science and Technology of China, Hefei 230026, People’s Republic of China

## Abstract

The title compound, C_28_H_20_O_4_, was synthesized by a bimolecular Zn–HCl reduction in glacial acetic acid using the meth­oxy-substituted xanthone as a starting material. The crystal structure shows that the 2,2′-meth­oxy­bixanthenyl­idene unit is an *E*-type conformation *anti*-folded conformer. The mol­ecule lies on an inversion center. The meth­oxy group is almost coplanar with the attached benzene ring, with a C—O—C—C torsion angle of 179.38 (14)°.

## Related literature

For background to dixanthylidenes, see: Korenstein *et al.* (1976 ▶); Agranat & Tapuhi (1979 ▶); Mao *et al.* (2011 ▶). For related structures, see: Mills & Nyburg (1963 ▶); Shi *et al.* (2012 ▶).
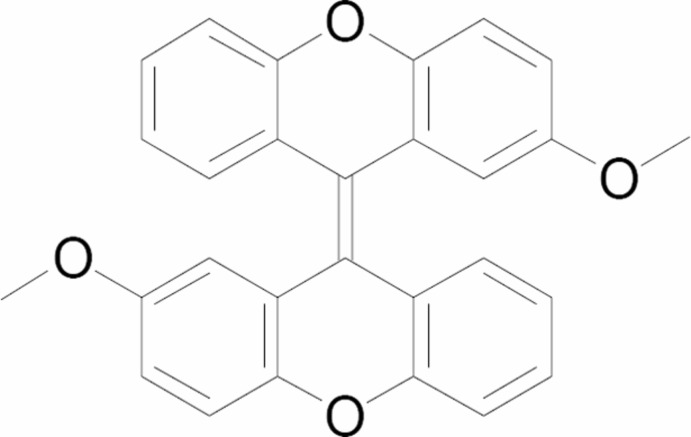


## Experimental

### 

#### Crystal data

C_28_H_20_O_4_
*M* *_r_* = 420.28
Monoclinic, 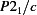

*a* = 8.5699 (7) Å
*b* = 7.5200 (6) Å
*c* = 16.6101 (18) Å
β = 102.682 (7)°
*V* = 1044.33 (16) Å^3^
*Z* = 2
Mo *K*α radiation
μ = 0.09 mm^−1^
*T* = 291 K
0.42 × 0.40 × 0.32 mm

#### Data collection

Oxford Diffraction Gemini S Ultra diffractometer
Absorption correction: multi-scan (*CrysAlis PRO*; Oxford Diffraction, 2007) ▶ *T*_min_ = 0.964, *T*_max_ = 0.972
6419 measured reflections
2205 independent reflections
1264 reflections with *I* > 2σ(*I*)
*R*_int_ = 0.029

#### Refinement

*R*[*F* ^2^ > 2σ(*F* ^2^)] = 0.037
*wR*(*F* ^2^) = 0.060
*S* = 1.00
2205 reflections
146 parameters
H-atom parameters constrained
Δρ_max_ = 0.16 e Å^−3^
Δρ_min_ = −0.17 e Å^−3^

### 

Data collection: *CrysAlis PRO* (Oxford Diffraction, 2007 ▶); cell refinement: *CrysAlis PRO*; data reduction: *CrysAlis PRO*; program(s) used to solve structure: *SHELXS97* (Sheldrick, 2008 ▶); program(s) used to refine structure: *SHELXL97* (Sheldrick, 2008 ▶); molecular graphics: *ORTEP-3 for Windows* (Farrugia, 2012 ▶); software used to prepare material for publication: *SHELXL97*.

## Supplementary Material

Crystal structure: contains datablock(s) global, I. DOI: 10.1107/S1600536813017297/zq2201sup1.cif

Structure factors: contains datablock(s) I. DOI: 10.1107/S1600536813017297/zq2201Isup2.hkl

Supplementary material file. DOI: 10.1107/S1600536813017297/zq2201Isup3.cml

Additional supplementary materials:  crystallographic information; 3D view; checkCIF report

## Figures and Tables

**Fig. 1.**
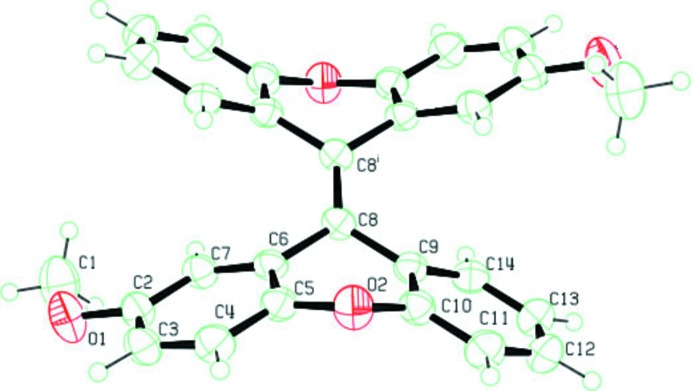
The structure of the title compound, showing 50% probability displacement ellipsoids and the atom-numbering scheme (symmetry code: i = -x+1, -y+1, -z+1).

## supplementary crystallographic information

### Comment

The 2,2'-substituted bixanthenylidenes have two conformers (*Z*, *E*), which are unstable at room temperature (Korenstein *et al.*, 1976). *Z*, *E* isomerization, with a low activation energy of 2,2'-disubstituted bixanthenylidenes, occurs easily at room temperature, and the ratio of *Z*/*E* in solution depends on the bulkiness of the the 2 and 2' substituents (Agranat & Tapuhi, 1979), 1:1.13 for the title compound. The crystal structure of the title compound shows that the *E*-type conformer lies on an inversion center located in the middle of the C8=C8*^i^* double bond (symmetry code: i = -*x*+1, -*y*+1, -*z*+1). The methoxy group is almost coplanar with the phenyl ring with a C1—O1—C2—C3 torsion angle of 179.38 (14) degrees (Fig.1). For related structures, see: Mills & Nyburg (1963); Shi *et al.* (2012).

### Experimental

2-Methoxyxanthone (8.8 mmol) was refluxed overnight in oxalyl dichloride (10 ml). The excess of oxalyl dichloride was removed, and the residue was dissolved in freshly distilled *p*-xylene (30 ml). Activated Cu powder (3.81 g, 60.0 mmol) was added and refluxed for 7 h with occasional shaking. The reaction solution was filtered, and the filter was concentrated and crystallized to give the title compound (Mao *et al.*, 2011). The title compound was dissolved in 10 ml of THF, and then adding 2 ml of toluene to the solution. The mixture solution was placed in a open flask upon standing at 45–50° C for 5 days, single crystals appeared, and were separated from the solvent by decantation.

### Refinement

All hydrogen positions were calculated after each cycle of refinement using a riding model, with C—H = 0.93 Å and *U*_iso_(H) = 1.2*U*_eq_(C) for aromatic H atoms, and with C—H = 0.96 Å and *U*_iso_(H) = 1.5*U*_eq_(C) for methyl H atoms.

### Crystal data

**Table:**

C_28_H_20_O_4_	*F*(000) = 440
*M**_r_* = 420.28	*D*_x_ = 1.337 Mg m^−^^3^
Monoclinic, *P*2_1_/*c*	Mo *K*α radiation, λ = 0.71073 Å
Hall symbol: -P 2ybc	Cell parameters from 2041 reflections
*a* = 8.5699 (7) Å	θ = 2.7–29.1°
*b* = 7.5200 (6) Å	µ = 0.09 mm^−^^1^
*c* = 16.6101 (18) Å	*T* = 291 K
β = 102.682 (7)°	Block, colourless
*V* = 1044.33 (16) Å^3^	0.42 × 0.40 × 0.32 mm
*Z* = 2	

### Data collection

**Table:**

Oxford Diffraction Gemini S Ultra diffractometer	2205 independent reflections
Radiation source: Enhance (Mo) X-ray Source	1264 reflections with *I* > 2σ(*I*)
Graphite monochromator	*R*_int_ = 0.029
Detector resolution: 15.9149 pixels mm^-1^	θ_max_ = 26.7°, θ_min_ = 3.0°
ω scans	*h* = −10→7
Absorption correction: multi-scan (*CrysAlis PRO*; Oxford Diffraction, 2007)	*k* = −7→9
*T*_min_ = 0.964, *T*_max_ = 0.972	*l* = −21→21
6419 measured reflections	

### Refinement

**Table:**

Refinement on *F*^2^	0 restraints
Least-squares matrix: full	H-atom parameters constrained
*R*[*F*^2^ > 2σ(*F*^2^)] = 0.037	*w* = 1/[σ^2^(*F*_o_^2^) + (0.0079*P*)^2^ + 0.150*P*] where *P* = (*F*_o_^2^ + 2*F*_c_^2^)/3
*wR*(*F*^2^) = 0.060	(Δ/σ)_max_ < 0.001
*S* = 1.00	Δρ_max_ = 0.16 e Å^−^^3^
2205 reflections	Δρ_min_ = −0.17 e Å^−^^3^
146 parameters	

### Special details

**Table:**

Experimental. CrysAlisPro, Oxford Diffraction Ltd., Version 1.171.33.34d (release 27-02-2009 CrysAlis171 .NET) Empirical absorption correction using spherical harmonics, implemented in SCALE3 ABSPACK scaling algorithm.
Geometry. All e.s.d.'s (except the e.s.d. in the dihedral angle between two l.s. planes) are estimated using the full covariance matrix. The cell e.s.d.'s are taken into account individually in the estimation of e.s.d.'s in distances, angles and torsion angles; correlations between e.s.d.'s in cell parameters are only used when they are defined by crystal symmetry. An approximate (isotropic) treatment of cell e.s.d.'s is used for estimating e.s.d.'s involving l.s. planes.
Refinement. Refinement of *F*^2^ against ALL reflections. The weighted *R*-factor *wR* and goodness of fit *S* are based on *F*^2^, conventional *R*-factors *R* are based on *F*, with *F* set to zero for negative *F*^2^. The threshold expression of *F*^2^ > σ(*F*^2^) is used only for calculating *R*-factors(gt) *etc*. and is not relevant to the choice of reflections for refinement. *R*-factors based on *F*^2^ are statistically about twice as large as those based on *F*, and *R*-factors based on ALL data will be even larger.

### Fractional atomic coordinates and isotropic or equivalent isotropic displacement parameters (Å^2^)

**Table:**

	*x*	*y*	*z*	*U*_iso_*/*U*_eq_	
O1	0.37126 (14)	−0.03674 (16)	0.29797 (7)	0.0612 (4)	
O2	0.15571 (11)	0.62217 (15)	0.36978 (6)	0.0464 (3)	
C1	0.4953 (2)	−0.1274 (2)	0.35211 (12)	0.0720 (6)	
H1A	0.5140	−0.2396	0.3283	0.108*	
H1B	0.4654	−0.1471	0.4038	0.108*	
H1C	0.5912	−0.0572	0.3611	0.108*	
C2	0.32826 (18)	0.1287 (2)	0.32026 (10)	0.0419 (4)	
C3	0.20616 (18)	0.2107 (2)	0.26345 (10)	0.0471 (4)	
H3	0.1611	0.1537	0.2141	0.057*	
C4	0.15201 (17)	0.3756 (2)	0.27995 (10)	0.0453 (4)	
H4	0.0692	0.4298	0.2424	0.054*	
C5	0.22169 (16)	0.4607 (2)	0.35303 (9)	0.0381 (4)	
C6	0.34973 (16)	0.3863 (2)	0.40865 (9)	0.0335 (4)	
C7	0.39884 (17)	0.2148 (2)	0.39259 (9)	0.0374 (4)	
H7	0.4793	0.1586	0.4308	0.045*	
C8	0.42069 (14)	0.49291 (19)	0.48303 (9)	0.0331 (4)	
C9	0.29155 (16)	0.58847 (19)	0.51278 (9)	0.0340 (4)	
C10	0.16438 (17)	0.6537 (2)	0.45337 (10)	0.0389 (4)	
C11	0.04103 (17)	0.7507 (2)	0.47368 (11)	0.0484 (5)	
H11	−0.0405	0.7970	0.4326	0.058*	
C12	0.04121 (19)	0.7774 (2)	0.55581 (11)	0.0501 (5)	
H12	−0.0398	0.8441	0.5704	0.060*	
C13	0.16141 (18)	0.7056 (2)	0.61689 (11)	0.0466 (4)	
H13	0.1593	0.7207	0.6722	0.056*	
C14	0.28401 (16)	0.6117 (2)	0.59522 (10)	0.0397 (4)	
H14	0.3635	0.5626	0.6365	0.048*	

### Atomic displacement parameters (Å^2^)

**Table:**

	*U*^11^	*U*^22^	*U*^33^	*U*^12^	*U*^13^	*U*^23^
O1	0.0783 (9)	0.0499 (8)	0.0496 (8)	0.0048 (7)	0.0012 (6)	−0.0137 (7)
O2	0.0441 (7)	0.0518 (7)	0.0374 (7)	0.0114 (6)	−0.0043 (5)	0.0007 (6)
C1	0.0937 (16)	0.0533 (12)	0.0666 (14)	0.0160 (12)	0.0122 (12)	−0.0051 (12)
C2	0.0445 (10)	0.0415 (10)	0.0395 (11)	−0.0044 (8)	0.0088 (8)	−0.0064 (9)
C3	0.0441 (10)	0.0614 (12)	0.0319 (10)	−0.0077 (9)	−0.0003 (7)	−0.0105 (9)
C4	0.0365 (9)	0.0615 (12)	0.0336 (10)	0.0011 (9)	−0.0019 (7)	−0.0005 (9)
C5	0.0331 (9)	0.0438 (10)	0.0356 (10)	−0.0003 (8)	0.0032 (7)	−0.0019 (8)
C6	0.0263 (8)	0.0423 (10)	0.0295 (9)	−0.0025 (7)	0.0010 (6)	−0.0003 (8)
C7	0.0344 (9)	0.0429 (10)	0.0320 (9)	−0.0009 (8)	0.0012 (7)	−0.0002 (8)
C8	0.0326 (8)	0.0341 (9)	0.0299 (9)	0.0004 (7)	0.0007 (6)	0.0020 (7)
C9	0.0300 (9)	0.0343 (10)	0.0362 (10)	−0.0012 (7)	0.0039 (7)	0.0003 (8)
C10	0.0361 (9)	0.0398 (10)	0.0384 (11)	−0.0001 (8)	0.0027 (7)	−0.0014 (8)
C11	0.0351 (10)	0.0475 (11)	0.0581 (12)	0.0091 (8)	0.0003 (8)	0.0009 (10)
C12	0.0409 (10)	0.0473 (11)	0.0640 (14)	0.0060 (8)	0.0159 (9)	−0.0050 (10)
C13	0.0451 (11)	0.0496 (11)	0.0473 (11)	0.0002 (8)	0.0149 (9)	−0.0012 (9)
C14	0.0342 (9)	0.0430 (10)	0.0404 (11)	−0.0020 (8)	0.0047 (7)	0.0020 (9)

### Geometric parameters (Å, º)

**Table:**

O1—C2	1.3716 (18)	C6—C8	1.4865 (19)
O1—C1	1.4086 (19)	C7—H7	0.9300
O2—C5	1.3930 (17)	C8—C8^i^	1.357 (2)
O2—C10	1.3942 (17)	C8—C9	1.4918 (17)
C1—H1A	0.9600	C9—C10	1.3892 (19)
C1—H1B	0.9600	C9—C14	1.3960 (19)
C1—H1C	0.9600	C10—C11	1.3857 (19)
C2—C7	1.3816 (19)	C11—C12	1.379 (2)
C2—C3	1.390 (2)	C11—H11	0.9300
C3—C4	1.373 (2)	C12—C13	1.387 (2)
C3—H3	0.9300	C12—H12	0.9300
C4—C5	1.3861 (19)	C13—C14	1.3777 (18)
C4—H4	0.9300	C13—H13	0.9300
C5—C6	1.3878 (18)	C14—H14	0.9300
C6—C7	1.400 (2)		
			
C2—O1—C1	118.46 (14)	C2—C7—H7	119.9
C5—O2—C10	114.32 (12)	C6—C7—H7	119.9
O1—C1—H1A	109.5	C8^i^—C8—C6	125.28 (16)
O1—C1—H1B	109.5	C8^i^—C8—C9	124.96 (17)
H1A—C1—H1B	109.5	C6—C8—C9	109.71 (11)
O1—C1—H1C	109.5	C10—C9—C14	117.04 (13)
H1A—C1—H1C	109.5	C10—C9—C8	117.27 (13)
H1B—C1—H1C	109.5	C14—C9—C8	125.63 (13)
O1—C2—C7	124.57 (15)	C11—C10—C9	122.30 (15)
O1—C2—C3	115.21 (15)	C11—C10—O2	117.08 (14)
C7—C2—C3	120.22 (15)	C9—C10—O2	120.62 (13)
C4—C3—C2	120.15 (15)	C12—C11—C10	118.85 (15)
C4—C3—H3	119.9	C12—C11—H11	120.6
C2—C3—H3	119.9	C10—C11—H11	120.6
C3—C4—C5	119.50 (15)	C11—C12—C13	120.42 (15)
C3—C4—H4	120.2	C11—C12—H12	119.8
C5—C4—H4	120.2	C13—C12—H12	119.8
C4—C5—C6	121.49 (15)	C14—C13—C12	119.68 (16)
C4—C5—O2	117.49 (14)	C14—C13—H13	120.2
C6—C5—O2	121.00 (14)	C12—C13—H13	120.2
C5—C6—C7	118.14 (14)	C13—C14—C9	121.50 (15)
C5—C6—C8	117.06 (13)	C13—C14—H14	119.3
C7—C6—C8	124.75 (13)	C9—C14—H14	119.3
C2—C7—C6	120.30 (15)		
			
C1—O1—C2—C7	0.3 (2)	C5—C6—C8—C9	37.73 (17)
C1—O1—C2—C3	179.38 (14)	C7—C6—C8—C9	−139.75 (14)
O1—C2—C3—C4	179.07 (13)	C8^i^—C8—C9—C10	140.63 (19)
C7—C2—C3—C4	−1.8 (2)	C6—C8—C9—C10	−36.97 (18)
C2—C3—C4—C5	1.0 (2)	C8^i^—C8—C9—C14	−42.2 (3)
C3—C4—C5—C6	2.7 (2)	C6—C8—C9—C14	140.17 (15)
C3—C4—C5—O2	−175.69 (13)	C14—C9—C10—C11	5.3 (2)
C10—O2—C5—C4	147.12 (13)	C8—C9—C10—C11	−177.34 (14)
C10—O2—C5—C6	−31.24 (19)	C14—C9—C10—O2	−174.28 (13)
C4—C5—C6—C7	−5.4 (2)	C8—C9—C10—O2	3.1 (2)
O2—C5—C6—C7	172.91 (12)	C5—O2—C10—C11	−147.58 (13)
C4—C5—C6—C8	176.96 (12)	C5—O2—C10—C9	32.0 (2)
O2—C5—C6—C8	−4.7 (2)	C9—C10—C11—C12	−2.6 (2)
O1—C2—C7—C6	178.01 (13)	O2—C10—C11—C12	176.93 (15)
C3—C2—C7—C6	−1.0 (2)	C10—C11—C12—C13	−1.1 (2)
C5—C6—C7—C2	4.5 (2)	C11—C12—C13—C14	2.1 (2)
C8—C6—C7—C2	−178.03 (13)	C12—C13—C14—C9	0.8 (2)
C5—C6—C8—C8^i^	−139.87 (19)	C10—C9—C14—C13	−4.3 (2)
C7—C6—C8—C8^i^	42.7 (3)	C8—C9—C14—C13	178.55 (14)

Symmetry code: (i) −*x*+1, −*y*+1, −*z*+1.
